# Shared capacity limitations in auditory scene analysis and central attention: Evidence from auditory search in a dual-task paradigm

**DOI:** 10.3758/s13414-026-03297-6

**Published:** 2026-07-14

**Authors:** Florian Kattner, Samuel Conrad

**Affiliations:** Institute for Mind, Brain and Behavor, Health and Medical University, Olympischer Weg 1, 14471 Potsdam, Germany

**Keywords:** Auditory scene analysis, Perceptual organization, Auditory attention, Psychological refractory period, Locus-of-slack, Auditory search

## Abstract

Auditory scene analysis enables listeners to segregate and group a mixture of sounds into coherent streams, but it remains controversial to what extent this process depends on the availability of central attention. While previous research suggests that the guidance of visual attention may be independent of central capacity limitations, it remains unclear whether auditory selective attention can operate in parallel with central attention. Across two online experiments, it was tested whether attentional search in complex auditory scenes is limited in capacity and subject to the central processing bottleneck using a dual-task paradigm with the locus-of-slack method. Participants were first asked to identify target sounds within auditory scenes of varying set sizes (1–8 sounds), either as a single task or together with a visual discrimination task at variable stimulus onset asynchronies (SOAs). Results from single-task blocks revealed robust auditory set-size effects on response times and error rates, indicating capacity limitations and serial processing during auditory scene analysis. In the dual-task paradigm, auditory search times increased at short SOAs, demonstrating a psychological refractory period. Critically, the auditory set-size effects persisted, but were not attenuated at short SOAs, suggesting that auditory search time has not been absorbed into the slack due to the central processing bottleneck. In contrast to some evidence from the visual attention literature, these findings indicate that auditory scene analysis and central attention depend on shared capacity limitations, thus highlighting the importance of attentional control in complex listening situations.

Most acoustical environments – whether in a subway station or a concert hall – contain multiple sound sources and the acoustic energy arising from different sounds is combined to produce a single complex waveform. When this waveform reaches the listener’s ears, the auditory system must separate it into distinct streams in order to identify the individual objects that produced the sounds (Eramudugolla et al., 2008). Auditory scene analysis refers to the processes by which a complex acoustical stimulus is organized into auditory streams that enable the perception of discrete auditory objects such as a human voice, an approaching car, or a piano melody (Bregman, 1990). Auditory scene analysis requires both the *segregation* of sounds from the background (e.g., identification of a specific voice among many others, known as the ‘cocktail party problem’; Cherry, 1953) and the *grouping* of different sound properties into a coherent auditory stream (e.g., the tones that create a coherent melody played by multiple instruments in an orchestra). For instance, a sequence of pure tones with alternating frequencies (ABA) can be perceived either as a single, ‘galloping’ rhythm (ABA-ABA...) or as two separate low- and high-frequency streams (AAA... and BBB...), depending on the frequency difference between A and B and on the presentation rate of the sequence (Noorden, 1975). There is an ongoing debate on whether the perceptual grouping and segregation of sounds based on temporal, spectral, or location cues depends on attention (Ding & Simon, 2012; Shamma et al., 2011; Snyder et al., 2012). On the one hand, there are examples of auditory streaming that are independent of attention (e.g., stream segregation by sound repetition does not depend on attentional task load; Masutomi et al., 2016). On the other hand, auditory figure-ground segregation was found to be susceptible to manipulations of task load, suggesting that some aspects of auditory scene analysis require attention (Molloy et al., 2019). Similarly, the frequency separation required to perceive two separate streams with tone sequences of alternating frequencies was found to be much smaller when participants were actively listening to the pitch. In contrast, stream segregation was impaired when attention was diverted from the tone sequence to a secondary task (Carlyon et al., 2001, 2003; Cusack et al., 2004; Thompson et al., 2011). These results suggest that the formation of an auditory stream crucially depends on the availability of attentional resources. According to a hierarchical model of stream segregation, individual acoustical features (e.g., the frequency difference or temporal characteristics) can be used to segregate a sequence of sounds pre-attentively. However, attention is required for the build-up of a coherent stream or auditory object (Cusack et al., 2004). For instance, when listening to a talker while music is being played in the background, pre-attentive processes may enable the segregation of speech and music (based on spectral and temporal information), but as long as attention is focused on speech, additional segregation or grouping processes may be impaired for the unattended music stream thereby preventing perception of the constituent parts (e.g., the identification of different instruments).

**Fig. 1:**
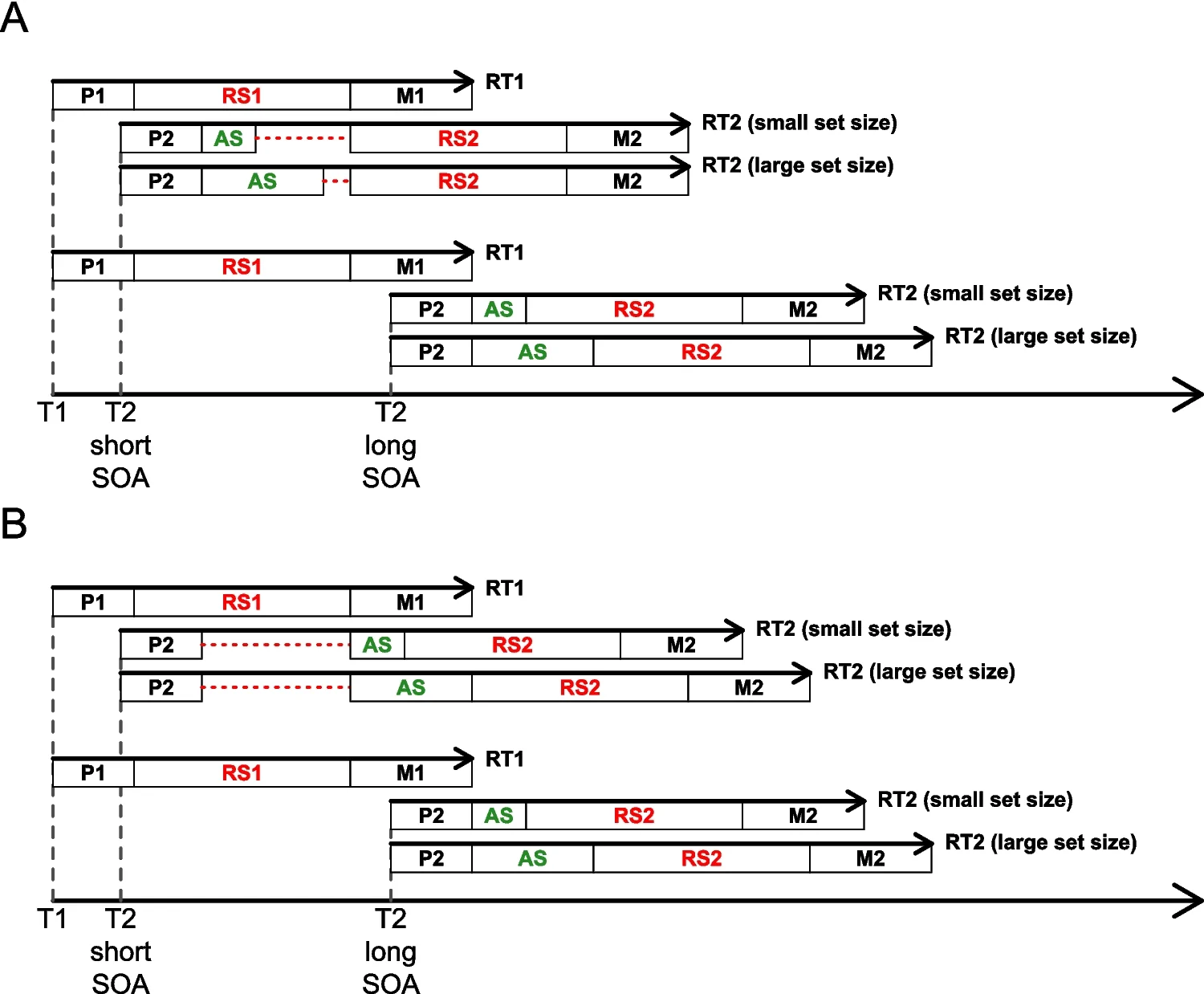
Illustration of the locus-of-slack method and the cognitive processes that sum up to the RT when assuming **(A)** distinct or **(B)** shared capacity limitations of central and auditory attention. *Note.*  T1 = Task 1 onset, T2 = Task 2 onset (short or long SOA), AS = Auditory Search (with small or large set size), P1/P2 = Perception (visual task 1, auditory task 2), RS1/RS2 = Response selection (task 1, task 2), M1/M2 = Motor response execution (task 1, task 2). **(A)** ***Distinct capacity limitations.*** Auditory search can be conducted in parallel with the response selection stage of task 1 and auditory search time is absorbed into the slack with short SOA, thus eliminating the auditory set-size effect (equal RT2 with small and large auditory set size). However, with long SOAs there will be no temporal overlap between the slack time and auditory search, leading to an auditory set-size effect (longer RT2 with large set size). **(B)** ***Shared capacity limitations.*** Auditory search cannot be conducted in parallel with response selection (of task 1) due to the central processing bottleneck, producing equivalent set-size effects with short and long SOAs (longer RT2 with large auditory set size)

The above findings also suggest that the capacity of auditory selective attention and thus the efficiency of auditory stream formation is limited. While most studies of auditory scene analysis used very simple sounds (e.g., pure tones), the capacity limitations of auditory attention have also been studied with more complex auditory scenes. For instance, participants can be presented with an ‘auditory search task’ (an analogue of visual search tasks to study the guidance of selective attention in visual scenes) that consists of a variable number of complex sounds (e.g., a human voice, a chicken, a siren, and a cello) and asked to localize a sound, identify a specific auditory object, or detect a change in the scene (Eramudugolla & Irvine, 2005; Eramudugolla et al., 2008; Kattner & Reimer, 2020; Lee, 2001). Using different versions of this task, it has been shown that both response times and error rates increase with the number of sounds in the scene (set size), indicating capacity limitations of auditory attention during auditory scene analysis. More specifically, the set-size effects suggest that the formation of discrete auditory objects during scene analysis requires attention (similar to the attention-dependent binding of features during visual conjunction search; e.g., Müller & Krummenacher, 2006; Nakayama & Silverman, 1986; Treisman & Gelade, 1980). Given the capacity limitations of auditory attention, attention needs to be shifted across the auditory scene in order to identify or localize a specific target sound, leading to sequential processing of the different objects in the scene. However, it remains unclear whether these attentional limitations during auditory scene analysis – as reflected in higher processing demands with more auditory objects – are due to a central processing bottleneck or whether auditory attention is independent of central attention.

The capacity limitations of central attention are most evident in dual-task situations, in which participants are asked to perform two tasks simultaneously (Pashler, 1994; Welford, 1952). It is typically observed that performance on the second task is impaired when the two tasks are presented in close temporal proximity, indicating a psychological refractory period (PRP) of central attention. More specifically, when the two tasks require fast and accurate responses, the response time to task 2 will be longer at short stimulus onset asynchronies (SOA) between the two tasks (Pashler, 1994; Schubert, 1999, 2008). This PRP effect can be explained by a central processing bottleneck model, which assumes that each task involves three sequential processes: stimulus perception, response selection, and motor response execution. While perception and motor response execution are generally assumed to be independent of central attention and can operate in parallel for the two tasks (at least when different stimulus and response modalities are involved), response selection is thought to depend on the availability of central attention. Therefore, the selection of responses for the two tasks must occur sequentially, thus inducing a bottleneck at the response selection stage, particularly with short SOAs (when the two response selection stages could overlap). Specifically, since the response selection for task 2 cannot start before response selection for task 1 has been completed, short SOAs (i.e., shorter than the response selection stage of task 1) induce an additional slack time, which will be added to the response time for task 2.

While the PRP effect suggests that response selection in dual-task situations occurs sequentially due to a central processing bottleneck, it is less clear whether the same attentional limitations are responsible for the sequential processing of auditory objects during auditory scene analysis. The locus-of-slack method can be used to assess whether a specific process of interest depends on the same capacity limitations of central attention that are responsible for the PRP effect (Schweickert, 1978, 1980). Therefore, the process of interest (e.g., auditory attention) needs to be part of task 2 in the dual-task paradigm and processing demands of this process need to be manipulated (e.g., by increasing set size in an auditory search task).

If the capacity limitations of auditory attention were independent of central attention, then the sequential processes of auditory scene analysis could operate in parallel with the central response selection stage of task 1. In this case, one would expect an underadditive interaction between auditory set size and SOA, with reduced or eliminated set-size effects at the short SOAs indicating that auditory search time can be absorbed into the slack time of the central response-selection bottleneck (i.e., in the delay of the response selection stage of task 2; see Fig. 1, panel A).

In contrast, if auditory stream formation depended on central attention (shared capacity limitations) and was subject to the central response selection bottleneck, then any increase in the demands on auditory attention should lead to a further (additive) increase in response times, because the auditory stream formation processes of task 2 cannot be performed in parallel with the response selection stage of task 1. In this case, manipulations of auditory set size and the SOA between task 1 and 2 should produce independent main effects on response times (i.e., larger set size should lead to longer response times with both short and long SOAs, see Fig. 1, panel B).

The locus-of-slack method has been applied to visual attention using a dual-task paradigm with an auditory discrimination task 1 and a visual conjunction search task 2 (Reimer & Schubert, 2019; Reimer et al., 2015). It has been found that, when response selection of task 1 is sufficiently complex (thus allowing for a long enough slack time), visual set-size effects were reduced at short SOAs, and the authors concluded that the attentional guidance required for visual conjunction search operates in parallel with the response selection stage of task 1 (Reimer & Schubert, 2019). That is, visual and central attention seem to rely on distinct capacity limitations.

There is currently only one study in which the locus-of-slack method was applied to auditory attention, providing some indication that auditory search may be independent of the central processing bottleneck (Kattner & Reimer, 2020). However, this was observed only in one of two experiments in which task 1 complexity was enhanced in order to increase the slack time at the response selection stage. In the first experiment of that study, an auditory search task 2 was combined with a relatively simple visual task 1 in which only two shapes were to be discriminated (circle and square). Here, auditory search time was found to depend on both the set size and the SOA between the two tasks, indicating capacity limitations of auditory and central attention. However, the set-size effect was not reduced at short SOAs, indicating that auditory search time has not been absorbed into the prolonged slack time at short SOAs. This could be due to either (a) auditory search being subject to the central processing bottleneck (shared capacity limitations) or (b) the slack time not being long enough for auditory search time to be absorbed (i.e., the response selection stage of task 1 could be finished before auditory search starts, see also Reimer & Schubert, 2019). This was tested in a second experiment with a slightly more complex visual discrimination task 1 (four different shapes), for which response selection should require more time. Interestingly, a significant interaction between set size and SOA was observed, suggesting that auditory attention has been operating in parallel with the prolonged response selection stage of task 1. Nevertheless, the overall evidence for auditory attention to be independent of central attention still is not entirely conclusive with only one experiment showing reduced set-size effects at short SOAs and another showing equivalent set-size effects across SOAs. This is because the statistical power of the study showing the SOA × set-size interaction may have been too small (Kattner & Reimer, 2020, Exp. 2), and the auditory set-size effects in both previous experiments of that study were relatively small in general compared to other studies using similar auditory search tasks (not using a dual-task paradigm, e.g., Eramudugolla et al., 2008; Lee, 2001). It has been discussed whether the discrepancy in set-size effects was due to the specific set of heterogeneous, very short, and rather artificial environmental sounds from various categories (250–400 ms, including animals, musical instruments, human noise, and artificial sounds). Lee (2001), for example, presented more homogeneous ‘auditory scenes’ consisting of spoken digits and letters, and Eramudugolla et al. (2008) presented more realistic and longer sounds including various musical instruments, voices, birds, and sirens. It is therefore important to provide additional, independent evidence for a possible modulation of auditory set-size effects in dual-task situations, using different stimulus materials, before concluding that auditory and central attention are subject to distinct capacity limitations.

The aim of the present study is therefore to revisit the question of whether (a) auditory scene analysis is subject to attentional capacity limitations and (b) these limitations of auditory attention rely on the central processing bottleneck. First, to test the capacity limitations of auditory attention, participants were presented with an auditory search task (identification of specific targets in an auditory scene), and the size of the auditory scene was manipulated from 1 to 8 different sounds. Any set-size-related increase in response times or error rates would indicate serial processing due to attentional capacity limitations during auditory scene analysis. Second, the same auditory search task is used in a dual-task paradigm with the auditory search task 2 starting at a variable SOA after a visual task 1. If auditory scene analysis was subject to the same central processing bottleneck as the PRP effect, then any increase in auditory set size should lead to an increase in the response times of task 2, regardless of the SOA. However, if the capacity limitations of auditory scene analysis were independent of the central processing bottleneck, then the set-size effect should be attenuated or eliminated at short SOAs, because auditory search time could be absorbed into the slack time that arises with short SOAs due to the bottleneck at the central response selection stage of task 1 (see Fig. 1).

Two experiments are presented using a similarly demanding visual discrimination task 1 as in Experiment 2 of Kattner and Reimer (2020) in order to provide sufficient slack time for some auditory search time to be absorbed. However, to induce larger and more reliable auditory set-size effects on response times and error rates, different auditory scenes are presented that consist of more homogeneous sounds from a single category (animal sounds). In Experiment 1, the sounds are taken from the same database of brief environmental (and rather artificial) sounds as in Kattner and Reimer (2020), but in Experiment 2 a different stimulus set of longer and more naturalistic recordings of animal vocalizations is used (providing more time for the build-up of auditory streaming). Finally, to quantify the set size effects in the absence of the dual-task paradigm, there will be a separate single-task block with only the auditory search task presenting the same auditory scenes as in the dual-task block (note that in Kattner & Reimer, 2020, there was only a short single-task practice block for both visual and auditory tasks). Thereby, the auditory set-size effects observed in the dual-task paradigm can be contrasted with the respective set-size effects under single-task conditions, thus providing an additional measure of the dual-task costs on auditory selective attention.

## Experiment 1

### Method

#### Participants

A power analysis was conducted (using the {WebPower} package for R) based on the previously observed effect size of the set-size effect on response times in an auditory search task (f= 0.4, $\eta ^2_p=$ 0.14^1^, Kattner & Reimer, 2020). It was estimated that a sample of 80 participants is required to observe an analog set-size effect in a repeated-measures ANOVA with moderately strong statistical power ($1-\beta =.89; \alpha =.05$).

A total of *N* = 80 participants (63 female, 16 male; 76 right-handed, four left-handed) were recruited from the student pool at Health and Medical University Potsdam. Ages ranged between 18 and 38 (*M* = 21.74, *SD* = 3.61). All participants reported normal or corrected-to-normal vision and no hearing loss. Participants were compensated with course credit.

#### Apparatus

The experiment was conducted as an online study, and the routines were programmed in PsyToolkit 3.4.0 (Stoet, 2010, 2017) and hosted on the European server (https://www.psytoolkit.org/c/3.6.2/). All participants were instructed to use headphones to run the experiment, and *n* = 9 and *n* = 34 reported to use ear-buds or in-ear headphones, whereas *n* = 8 and *n* = 29 reported to use on-ear or over-ear headphones, respectively.

**Fig. 2:**
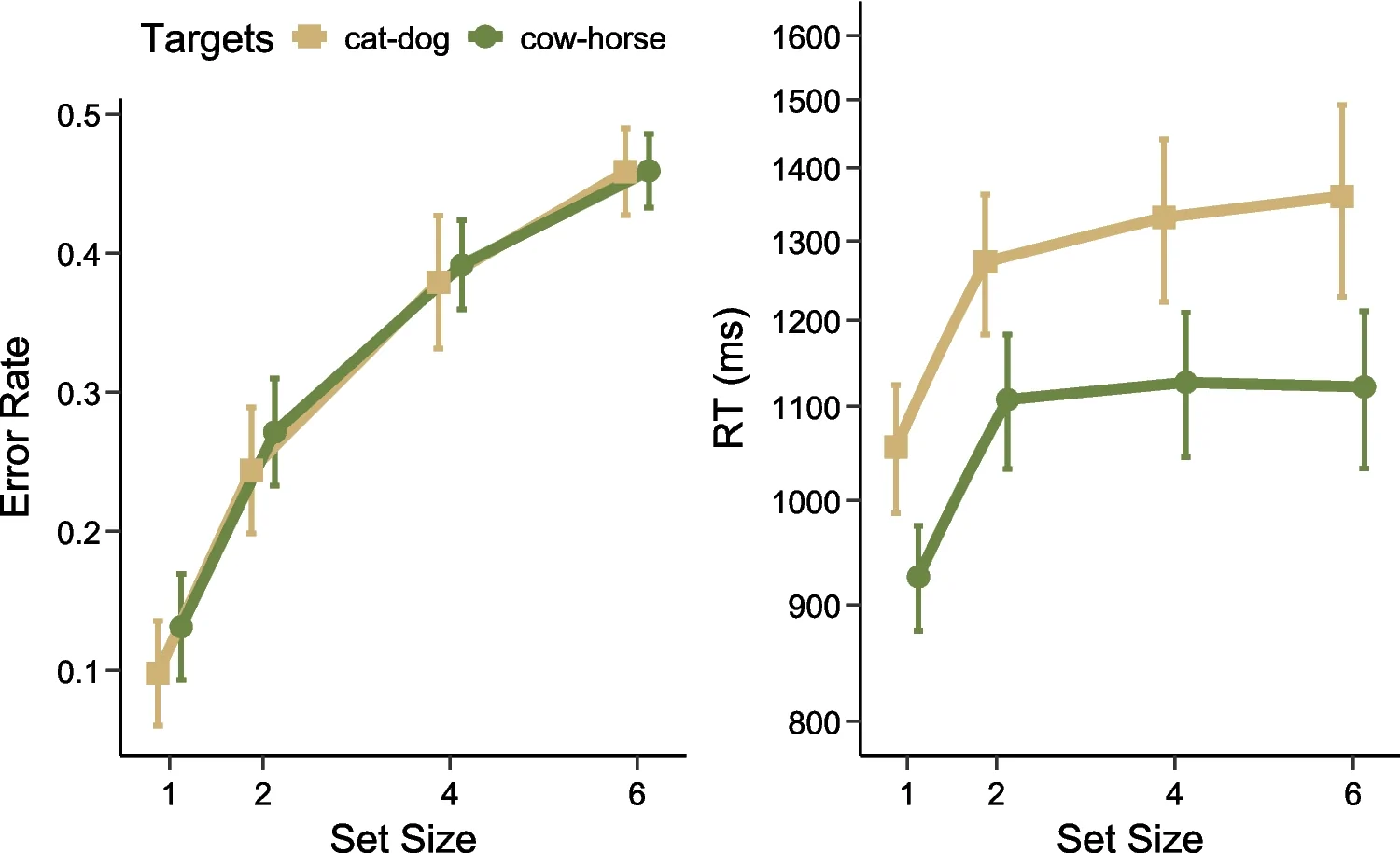
Error rates and response times during single-task auditory search in a scene with 1, 2, 4, or 6 different sounds. *Note.* The task was to discriminate between cat and dog (*green lines*) or cow and horse (*yellow lines*). *Error bars* indicate one standard error of the mean above and below the mean

#### Stimuli

Eight unique animal sounds (cat meowing, cow, dog, frog, horse snort, pig grunt, raven, and sheep) were selected from a database of brief environmental sounds (Fabiani et al., 1996). Each monophonic sound was cut to a duration of 200 ms, including 10-ms rise and fall times (11.025-kHz sampling rate). The original durations of the eight selected sounds varied between 214 and 395 ms (M=297;SD=74 ms) and the peak frequency varied between 180 Hz (frog) and 1372 Hz (raven; M=517.75;SD=415.24 Hz). Two different sets of 288 auditory scenes each were created by mixing either one, two, four or six different animal sounds (72 scenes per set size). The sounds within each auditory scene were played synchronously, and the total duration of each scene was also 200 ms. The scenes of set size 1 all contained either the dog or the cat as the target sound, whereas the scenes of set 2 contained either the cow or the horse as the target sound. To allow for spatial separation of the animal sounds (when using headphones), unique interaural level differences (left/right amplitude ratios 0/1 - 1/0) and interaural time differences (18–69 ms) were assigned randomly to each individual sound of an auditory scene.

#### Design and procedure

The study comprised three main parts: the headphone screening test, the single-task auditory search (to assess the capacity limitations of auditory attention), and the dual-task paradigm (to test whether auditory search depends on the capacity limitations of central attention).

Participants started with the *headphone screening test* (based on Woods et al., 2017) to ensure they were using headphones rather than loudspeakers (to allow for spatial segregation of sounds based on interaural time and level differences). Prior to the screening test, participants were presented with continuous pink noise and instructed to adjust the volume of their computer and headphones to a ‘comfortable’ level. During the test, three 200-Hz stereo pure tones were presented successively and together with a blue box on the screen for 1 s each and with 200-ms inter-stimulus intervals between the tones. The sound pressure level of one tone was 6 dB lower than that of the other two tones. For one of the two tones with the higher level, the phase was reversed between the left and right channels, which reduces the sound pressure level when presented via loudspeakers due to acoustic interference, but not when presented via headphones. The participants’ task was to indicate which tone was ‘softer’ than the others by clicking on the respective blue box. The test was passed if five or six out of six responses in a row were correct. The screening ended when the test was passed or after 36 trials, whichever came first. If participants did not pass the test, a message appeared on the screen indicating that the audio system was insufficient to run the experiment. Participants were allowed to restart the experiment using headphones.

If the headphone screening was passed, the study continued with the *single-task auditory search*. At the beginning of each trial, a blue shrinking fixation rectangle was presented for 950 ms (on a black screen). Then the auditory scene was presented together with a short text prompt asking participants to press the A or S key on the keyboard to indicate whether the scene contained target animal 1 or 2 (for *n* = 53 participants the target sounds were a mooing cow or a neighing horse, and for *n* = 26 participants the target sounds were a barking dog or a meowing cat). Participants were instructed to respond as quickly and as accurately as possible. The response deadline was 5000 ms. After the response, a short text feedback was presented for 1000 ms, indicating whether the response was correct (green font color) or wrong (red font color). There were 20 practice trials (with randomly selected auditory scenes), followed by 288 experimental trials, with each unique auditory scene being presented once.

Participants then continued with the *dual-task* paradigm. Again, the beginning of each trial was indicated by a shrinking blue fixation rectangle (950 ms), which was followed by a grey T presented in the center of the black screen for 50 ms. The T was either upright, upside down, tilted to the left, or tilted to the right. The auditory scene was presented after a variable SOA of 50, 100, 350, or 800 ms (relative to the onset of the central T). Participants were asked to first indicate the orientation of the T with the arrow keys (task 1) and then indicate whether target animal 1 or 2 was contained in the auditory scene (task 2). The same target animals were used as in the auditory search single task. The response deadline for the auditory search task was 6000 ms. When both responses were given, feedback was presented indicating whether the correct or incorrect animal sound was detected, or whether the response was invalid (when task 1 was incorrect or when no response was given within the response deadline). There were 20 dual-task practice trials (randomly selected auditory scenes), followed by 288 experimental dual-task trials with a unique auditory scene on each trial.

### Results

#### Single-task auditory search

Figure 2 illustrates the response times and error rates in *single-task auditory search*. As can be seen, both increased monotonically with the number of sounds in the auditory scene (set size). This observation was confirmed by significant main effects of set size on error rates, F(2.34,182.65)=272.01, $\textit{MSE} = 0.01$, p<.001, $\hat{\eta }^2_G = .534$, and response times F(1.43,111.37)=59.60, $\textit{MSE} = 33,270.36$, p<.001, $\hat{\eta }^2_G = .098$, respectively. However, while error rates increased significantly from set size 1 to 2, t(78)=14.61, $p_\mathrm {\scriptstyle BH(3)} < .001$, 2 to 4, t(78)=10.19, $p_\mathrm {\scriptstyle BH(3)} < .001$ and 4 to 6, t(78)=6.53, $p_\mathrm {\scriptstyle BH(3)} < .001$, there was a significant increase in response times only from set size 1 to 2, t(78)=11.77, $p_\mathrm {\scriptstyle BH(3)} < .001$, but not from 2 to 4, t(78)=2.48, $p_\mathrm {\scriptstyle BH(3)} = .023$, and 4 to 6, t(78)=0.49, $p_\mathrm {\scriptstyle BH(3)} = .628$ (alpha-corrected contrasts adjusted for three planned comparisons).

#### Dual-task auditory search

**Fig. 3:**
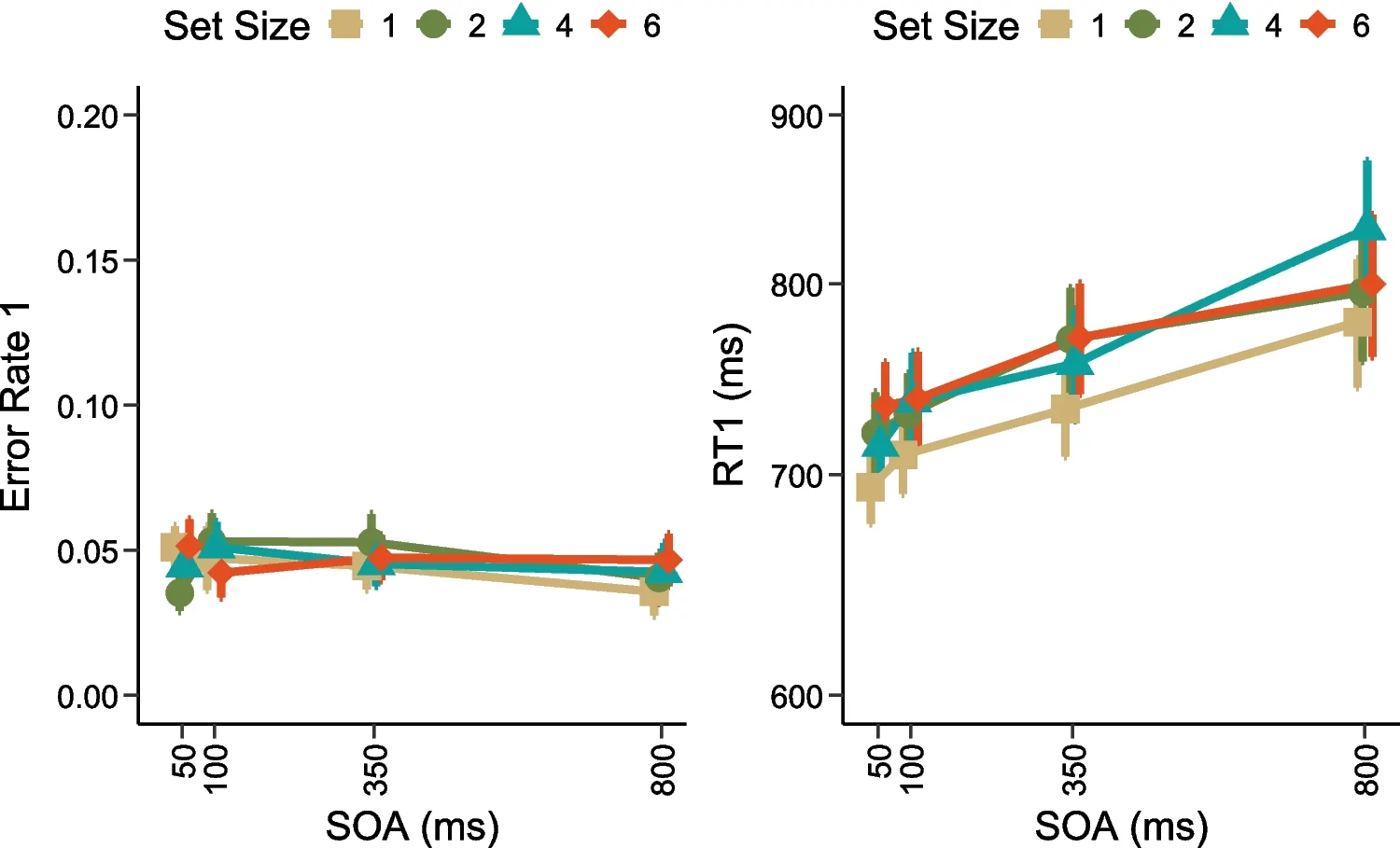
Error rates and response times in the visual discrimination task 1 of the dual-task paradigm as a function of SOA and auditory set size (in task 2). *Note.* *Error bars* depict one standard error of the mean above/below the mean

The error rates and response times in *task 1* (visual orientation discrimination) of the dual-task paradigm are illustrated in Fig. 3. As can be seen, the *error rates* did not differ as a function of the SOA, F(2.87,223.79)=1.16, $\textit{MSE} = 0.00$, p=.325, $\hat{\eta }^2_G = .001$, and the set size of the auditory scene in task 2, F(2.75,214.63)=0.14, $\textit{MSE} = 0.00$, p=.923, $\hat{\eta }^2_G = .000$. There was also no interaction, F(6.64,517.60)=1.29, $\textit{MSE} = 0.00$, p=.258, $\hat{\eta }^2_G = .003$. However, there was an increase in *response times* of task 1 with both the SOA between the tasks, F(1.22,94.89)=12.79, $\textit{MSE} = 87,253.05$, p<.001, $\hat{\eta }^2_G = .017$, and the set size of the auditory scene, F(2.03,158.25)=9.05, $\textit{MSE} = 11,561.18$, p<.001, $\hat{\eta }^2_G = .003$, possibly indicating some response grouping (i.e., delaying the response to task 1 until the auditory search task 2 has been processed). To assess the contribution of response grouping, the data were also analyzed as a function of the delay between the two responses (inter-response times; see below). The analysis revealed no interaction of SOA and set size on task 1 response times, F(6.79,529.73)=2.59, $\textit{MSE} = 4,689.36$, p=.013, $\hat{\eta }^2_G = .001$.

**Fig. 4:**
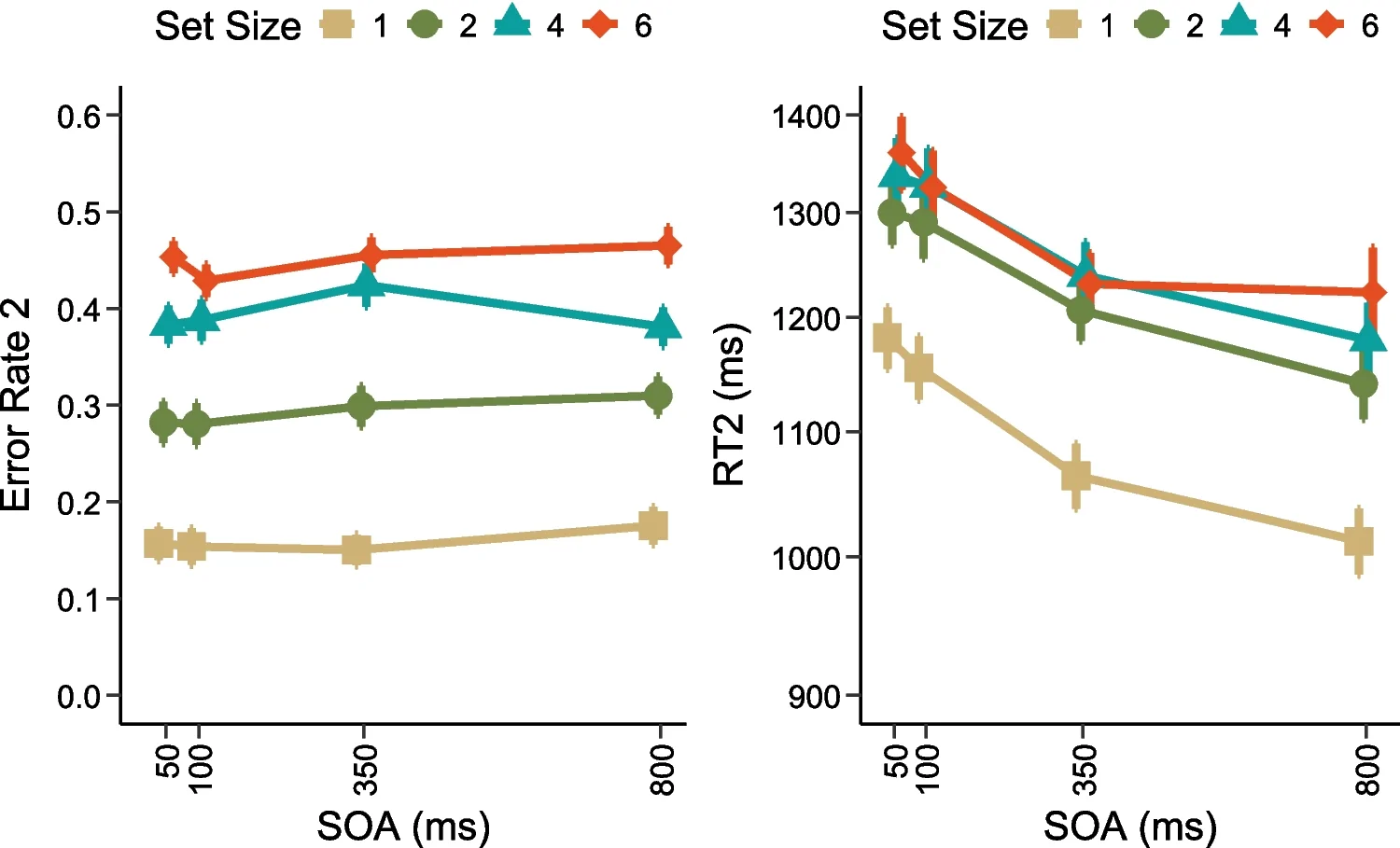
Error rates and response times in the auditory search task 2 of the dual-task paradigm as a function of SOA, set size, and target presence in the auditory scene. *Note.* *Error bars* depict one standard error of the mean above and below the mean

**Fig. 5:**
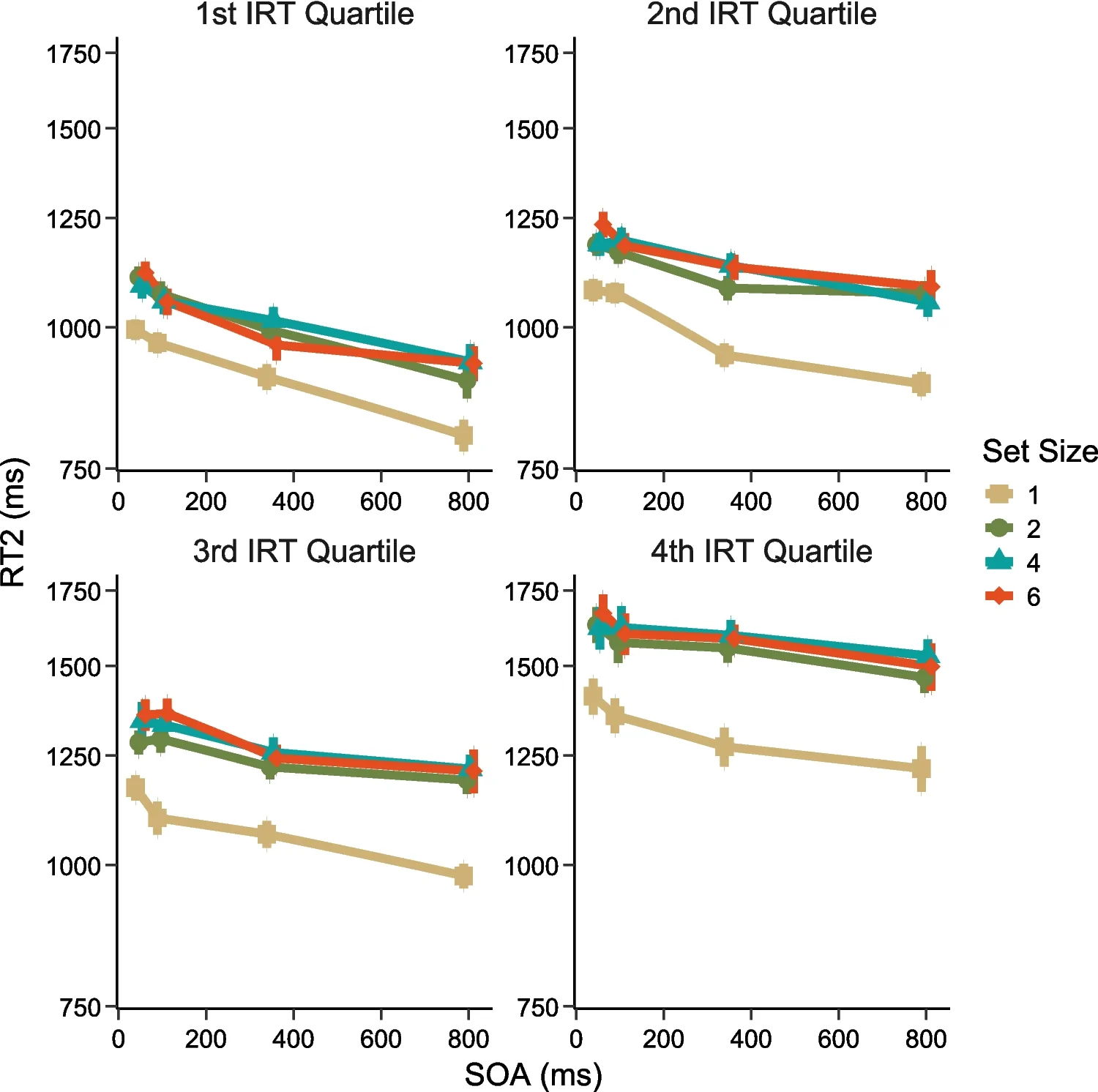
IRT analysis showing similar auditory set-size effects, regardless of SOA, with short and long IRTs in Experiment 1

Figure 4 illustrates the proportion of errors and the response times in the auditory search *task 2* of the dual-task paradigm. As can be seen, there were remarkable effects of set size on both error rates, F(2.42,188.69)=207.04, $\textit{MSE} = 0.03$, p<.001, $\hat{\eta }^2_G = .296$, and response times, F(1.54,119.92)=59.23, $\textit{MSE} = 71,380.88$, p<.001, $\hat{\eta }^2_G = .057$, indicating attentional capacity limitations during auditory scene analysis – which appear to be even more pronounced than under single-task auditory search conditions. Planned contrasts (adjusted for multiple comparisons according to Benjamini & Hochberg, 1995) revealed that error rates in the auditory search task increased from set size 1 to 2, t(78)=12.21, $p_\mathrm {\scriptstyle BH(3)} < .001$, 2 to 4, t(78)=9.96, $p_\mathrm {\scriptstyle BH(3)} < .001$, and 4 to 6, t(78)=5.16, $p_\mathrm {\scriptstyle BH(3)} < .001$. Likewise, response times increased from set size 1 to 2, t(78)=9.75, $p_\mathrm {\scriptstyle BH(3)} < .001$, and from 4 to 6, t(78)=2.01, $p_\mathrm {\scriptstyle BH(3)} = .048$, whereas the contrast between set size 2 and 4 was not statistically significant, t(78)=3.14, $p_\mathrm {\scriptstyle BH(3)} = .004$. Moreover, there was also a significant SOA effect on response times, F(2.12,165.38)=64.45, $\textit{MSE} = 37,531.94$, p<.001, $\hat{\eta }^2_G = .046$, indicating capacity limitations of central attention due to the bottleneck at the response selection stage. The SOA effect on error rates failed to reach statistical significance, F(2.82,219.67)=2.26, $\textit{MSE} = 0.01$, p=.087, $\hat{\eta }^2_G = .003$. Importantly, there was no interaction between SOA and set size both for error rates, F(7.68,598.68)=1.50, $\textit{MSE} = 0.01$, p=.156, $\hat{\eta }^2_G = .004$, and response times, F(6.53,509.07)=1.31, $\textit{MSE} = 12,780.59$, p=.249, $\hat{\eta }^2_G = .001$, suggesting that the auditory search process was also subject to the central processing bottleneck and the search time could not be absorbed into the slack (i.e., the set-size effect did not decrease at short SOAs).

To test whether the effects of auditory set size were influenced by strategic response grouping (postponing the response to task 1 until task 2 was completed), auditory search times were analyzed as a function of the *inter-response time (IRT)*, with short IRTs indicating response grouping (IRT = RT2-RT1+SOA, see Kattner & Reimer, 2020). Therefore, IRTs were binned into quartiles for each participant and SOA × set-size condition, and the IRT quartile used as an additional within-subjects factor in a 4 (IRT quartile) × 4 (SOA) × 4 (set size) repeated-measures ANOVA of task 2 response time. In addition to the main effects of set size and SOA, the analysis revealed a significant main effect of the IRT quartile, F(1.10,84.74)=166.24, $\textit{MSE} = 1,293,510.49$, p<.001, $\hat{\eta }^2_G = .277$, with shorter auditory search times with short IRTs (see Fig. 5). There was no significant interaction between SOA and IRT quartile, F(5.35,411.71)=1.85, $\textit{MSE} = 42,604.24$, p=.097, $\hat{\eta }^2_G = .001$. However, there was a significant interaction between set size and IRT quartile, F(4.80,369.47)=10.81, $\textit{MSE} = 50,777.26$, p<.001, $\hat{\eta }^2_G = .004$, indicating that auditory search times may have been affected by response grouping to some extent, leading to smaller set-size effects at short IRTs due to postponed task 1 responses. Nevertheless, the analysis revealed no three-way interaction between SOA, set size and IRT quartile, F(10.43,803.41)=0.78, $\textit{MSE} = 50,665.70$, p=.657, $\hat{\eta }^2_G = .001$, with the set-size effects being largely equivalent across different SOAs, regardless of the IRT, suggesting that sequential processing of auditory and central attention was not contingent on response grouping.

### Discussion

Experiment 1 demonstrated clear capacity limitations of auditory attention with relatively large effects of the set size of auditory scenes on both error rates and response times. This was observed both under single-task and dual-task conditions (e.g., increases in response times of about 200 ms from set size 1 to set size 6). The magnitude of auditory set-size effects is comparable to previous single-task auditory search studies using similar paradigms (Eramudugolla et al., 2008; Lee, 2001), but much larger compared to the set-size effects of 20 ms in the previous dual-task study (Kattner & Reimer, 2020).

Moreover, applying the auditory search task in a dual-task paradigm revealed that auditory search may be subject to a central processing bottleneck at the response selection stage. Specifically, at the very short SOAs – which are associated with longer auditory search times due to the central processing bottleneck (PRP effect) – the effect of set size was largely equivalent to the set-size effect at longer SOAs, indicating that no auditory search time has been absorbed into the slack time due to the central bottleneck. This suggests that the auditory attention is subject to the capacity limitations of central attention, which account for the processing bottleneck at the response selection stage. This finding clearly deviates from the previously reported reduction of the set-size effect at short SOAs (Kattner & Reimer, 2020, Exp. 2), which may have been an artifact due to the very small magnitude of the auditory set-size effect to begin with. It is therefore important to replicate the finding in a second experiment using slightly different stimulus materials.

Admittedly, the error rate in the auditory search task of Experiment 1 was relatively high, increasing with the set size from about 10% to about 45% under both single and dual-task conditions. This suggests that the task to identify a target within the short presentations of auditory scenes (<500 ms) may have been extremely challenging. Moreover, the short duration of the animal sounds may not allow for a complete build-up of auditory streaming. Specifically, since the auditory system may need to accumulate evidence for multiple sound sources over time, listeners will initially perceive temporal coherence (one stream), and the probability of stream segregation increases with longer sound duration (e.g., Anstis & Saida, 1985; Bregman, 1978). We therefore decided to replicate the findings with new auditory scenes consisting of longer and more realistic sounds (2 s), possibly allowing for better spatial, temporal, and spectral separation of the different sound sources.

## Experiment 2

To conceptually replicate the findings of Experiment 1, a second experiment was conducted using slightly different auditory scenes (using set sizes between two and eight animal sounds), longer sound durations (2 s) and a modified auditory search task, asking participants to identify the presence or absence of a single target sound (rather than discriminating two possible target sounds as in Experiment 1). Moreover, a slightly more demanding visual 4-choice discrimination is used as task 1 in Experiment 2 in order to further test the response selection stage. This allows us to test whether the absence of a modulation of the set-size effect by SOA in Experiment 1 may have been due to the slack time being too short for auditory search time to be absorbed. Therefore, the orientation discrimination task 1 was replaced by a two-dimensional shape-and-color discrimination task 1 in Experiment 2.

### Method

#### Participants

A total of *N*=71 student participants (55 female, 14 male, 2 non-binary; 67 right-handed, four left-handed) were recruited from the student pool at Health and Medical University Potsdam and Medical School Berlin. Ages ranged between 18 and 42 years (*M*=22.34, *SD*=4.42). All participants reported normal or corrected-to-normal vision and no hearing loss. Participants were compensated with course credit via the respective school’s Sona system.

**Fig. 6:**
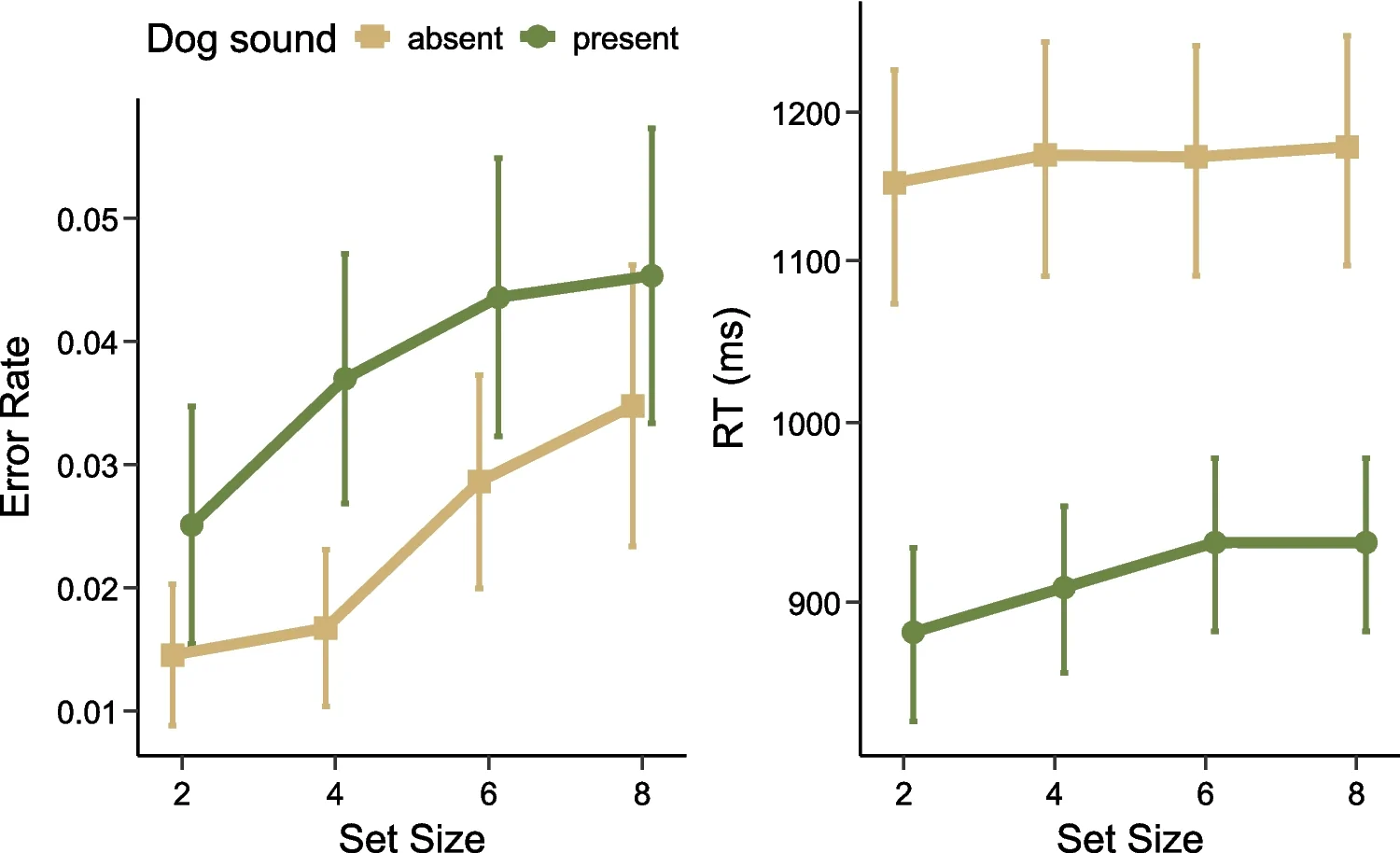
Error rates and response times during single-task search in an auditory scene with 2, 4, 6, or 8 different sounds in Experiment 2. *Note.* The auditory search task was to indicate whether or not the target sound (barking dog) was present in the auditory scene. *Error bars* indicate one standard error of the mean above and below the mean

#### Apparatus and stimuli

The experiment was again programmed as an online study in PsyToolkit 3.4.0 (Stoet, 2010, 2017) and participants were instructed to use headphones to run the experiment. As in Experiment 1, this was tested with a headphone screening test (Woods et al., 2017) which had to be passed in order to participate in the experiment. Based on the participants’ self-report, *n* = 5 used ear-buds, *n* = 25 used in-ear headphones, *n* = 7 used on-ear headphones, and *n* = 34 used over-ear headphones to run the tasks.

The auditory scenes for Experiment 2 were created analog to Experiment 1, but with ten slightly different and more realistic individual sounds of 2-s duration (bats, canary, cat, chicken, dog, donkey, duck, frog, owl, sheep). A total of 256 unique auditory scenes were created by mixing either two, four, six, or eight different animal sounds. Unique and randomly assigned interaural level differences (.1/.9 to .9/.1 left/right ratios) were used for each individual animal sound of a scene to allow for spatial separation. Half of the auditory scenes contained the target sound (dog), and the other half did not contain the target sound. In the scenes containing the target, the lateralization of the target (left/right) was counterbalanced. Thirty-two unique auditory scenes were created for each combination of set size and target presence, resulting in a total of 256 different scenes.

**Fig. 7:**
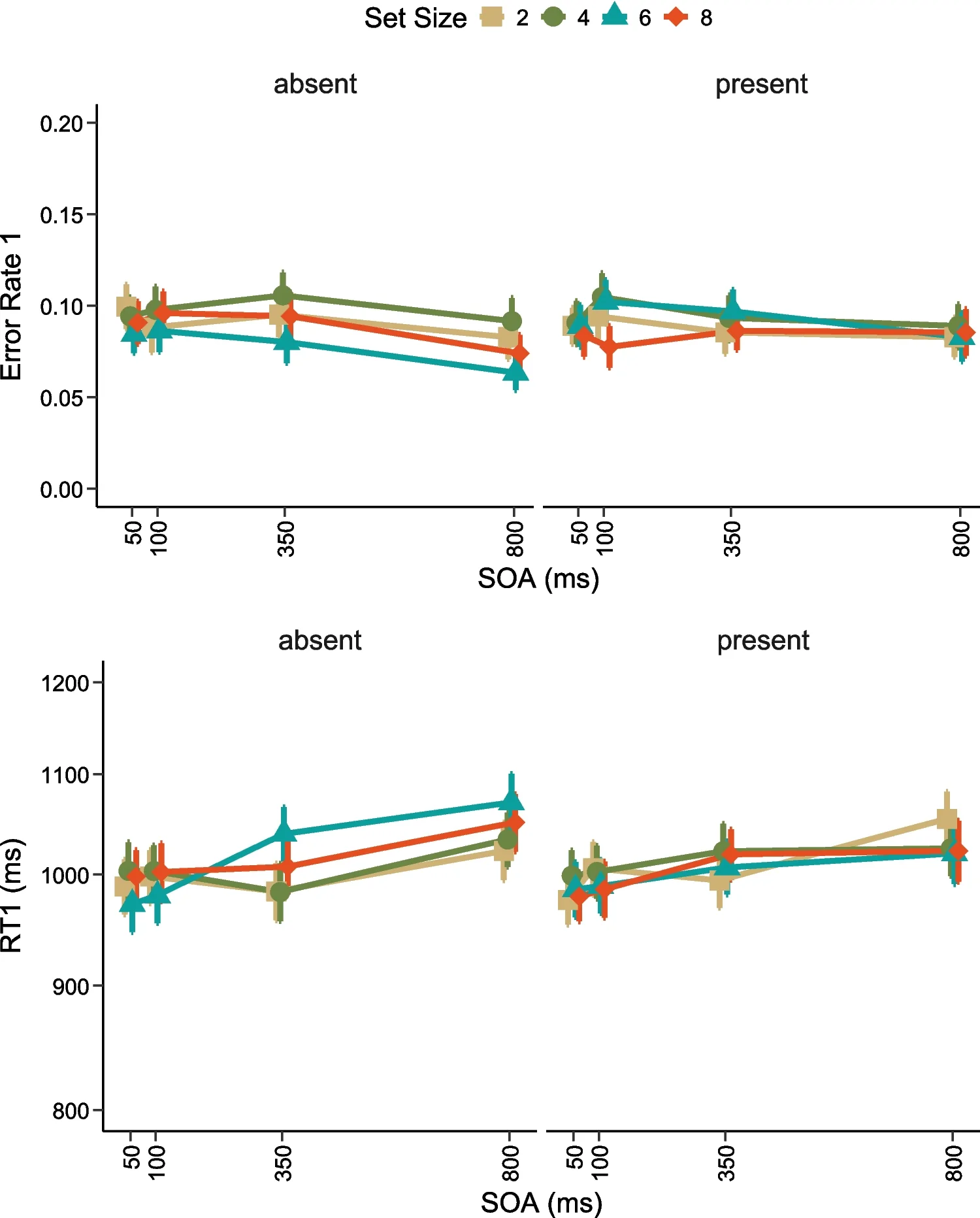
Error rates and response times in the visual discrimination task 1 of the dual-task paradigm in Experiment 2 as a function of the SOA, the set size, and the target presence in the auditory search task 2. *Note.* Task 1 (illustrated here) was a four-choice visual orientation discrimination task, and task 2 was an auditory search task in scenes with 2, 4, 6, or 8 auditory objects. *Error bars* depict one standard error of the mean above/below the mean

**Fig. 8:**
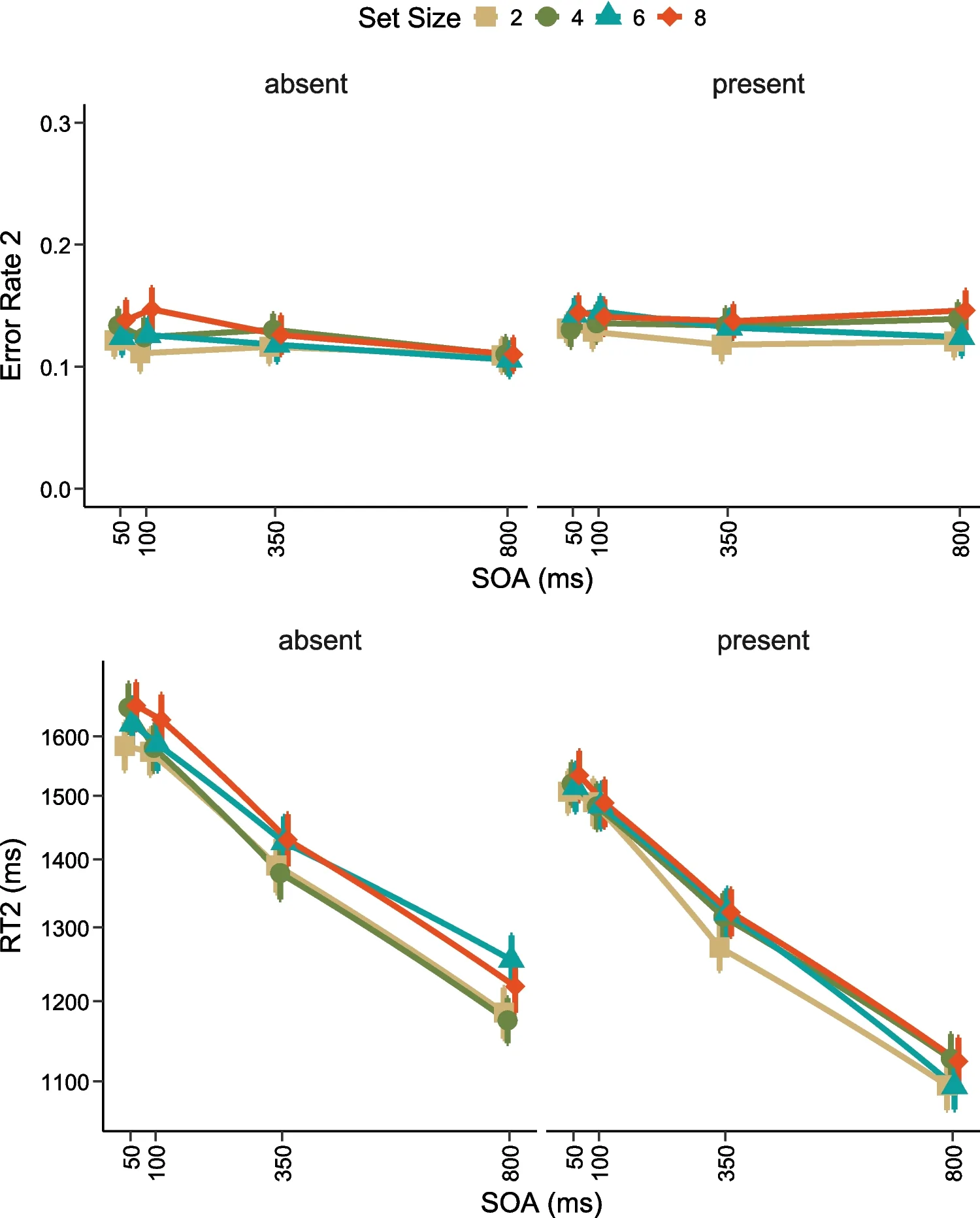
Error rates and response times in the auditory search task 2 of Experiment 2 as a function of SOA, set size, and target presence in the auditory scene. *Note.* Task 1 was a four-choice visual orientation discrimination task, and task 2 (illustrated here) was to indicate the presence of a target sound in scenes with 2, 4, 6, or 8 auditory objects. *Error bars* depict one standard error of the mean above and below the mean

#### Procedure

The procedure was essentially the same as in Experiment 1, comprising the headphone-screening test (Woods et al., 2017), a single-task block (256 trials) and a dual-task block (512 trials, with each auditory scene presented twice). There were twenty additional practice trials prior to both the single-task and the dual-task block. To prolong the response selection stage of task 1, a slightly more complex two-dimensional visual discrimination task was used in the dual-task paradigm of Experiment 2: Participants were asked to categorize the target as one of four possible targets based on color and shape (blue plus sign, blue cross, green plus sign, or green cross), by pressing the left, right, up or down arrow key. There were 20 practice trials to learn the respective stimulus-response association. Also in contrast to Experiment 1, the auditory search task required participants to indicate whether the target sound (dog) was present or absent in the auditory scene by pressing the A or S key, respectively. Participants were instructed to respond as quickly and accurately as possible. The response deadline for the auditory search task was 5000 ms in the single-task condition and 6000 ms in the dual-task condition.

### Results

#### Single-task auditory search

Figure 6 illustrates the response times and error rates in the single auditory search task of Experiment 2. As can be seen, both error rates and response times increased significantly with the number of sounds in the auditory scene, suggesting capacity limitations of auditory attention (i.e., serial processing of the different sounds in an auditory scene). A 4 (set size) × 2 (target presence) repeated-measures ANOVA confirmed significant set-size effects for errors, F(2.62,183.10)=11.50, $\textit{MSE} = 0.00$, p<.001, $\hat{\eta }^2_G = .035$, and for RTs, F(2.48,173.64)=15.61, $\textit{MSE} = 2,934.91$, p<.001, $\hat{\eta }^2_G = .003$. Planned contrasts revealed a non-significant increase in error rates from set size 2 to 4, t(70)=2.41, $p_\mathrm {\scriptstyle BH(3)} = .043$, 4 to 6, t(70)=2.23, $p_\mathrm {\scriptstyle BH(3)} = .043$, and 6 to 8, t(70)=1.06, $p_\mathrm {\scriptstyle BH(3)} = .292$. For response times, there was a significant increase from set size 2 to 4, t(70)=4.04, $p_\mathrm {\scriptstyle BH(3)} < .001$, but not from 4 to 6, t(70)=2.15, $p_\mathrm {\scriptstyle BH(3)} = .052$, and 6 to 8, t(70)=0.72, $p_\mathrm {\scriptstyle BH(3)} = .471$.

The ANOVA also revealed a significant main effect of target presence, with slower responses when the target was absent in the scene, F(1,70)=82.24, $\textit{MSE} = 110,118.44$, p<.001, $\hat{\eta }^2_G = .172$, indicating non-exhaustive auditory search in case of target presence. Target presence also affected the error rates, F(1,70)=17.57, $\textit{MSE} = 0.00$, p<.001, $\hat{\eta }^2_G = .028$, with more errors on target-present trials (see Fig. 6). However, the set-size effect did not differ between auditory scenes with and without the target, as confirmed by a non-significant interaction both for error rates, F(2.54,177.49)=0.97, $\textit{MSE} = 0.00$, p=.395, and response times, F(2.65,185.80)=3.20, $\textit{MSE} = 2,649.90$, p=.030.

#### Dual-task auditory search

Figure 7 illustrates the error rates and response times in the *visual discrimination task 1* of the dual-task paradigm. A 4 (SOA) × 4 (set size) × 2 (target presence) repeated-measures ANOVA revealed that the *error rate in task 1* was not affected significantly by the SOA, F(2.86,200.23)=2.39, $\textit{MSE} = 0.01$, p=.073, $\hat{\eta }^2_G = .002$, but there was a significant set size, F(2.96,207.39)=3.29, $\textit{MSE} = 0.00$, p=.022, $\hat{\eta }^2_G = .002$. Planned contrasts revealed a significant difference in task 1 error between set size 4 and 6 only, t(70)=-2.78, $p_\mathrm {\scriptstyle BH(3)} = .021$. There was no main effect of target presence (in the auditory scene of task 2) on task 1 errors though, F(1,70)=0.04, $\textit{MSE} = 0.00$, p=.849, and target presence did not interact with SOA, F(2.85,199.59)=0.78, $\textit{MSE} = 0.01$, p=.502, or set size, F(2.81,196.51)=2.24, $\textit{MSE} = 0.01$, p=.089. There was also no set size × SOA interaction, F(7.77,543.97)=0.42, $\textit{MSE} = 0.00$, p=.904, and no three-way interaction on task 1 error rate, F(7.61,532.50)=0.58, $\textit{MSE} = 0.01$, p=.789.

For the *response times of task 1*, there was a main effect of SOA, F(1.56,108.98)=16.78, $\textit{MSE} = 30,790.12$, p<.001, $\hat{\eta }^2_G = .007$, with a small increase in response times at the longer SOAs, possibly indicating response grouping. There was also a significant interaction, F(7.83,548.21)=2.71, $\textit{MSE} = 7,773.48$, p=.007, $\hat{\eta }^2_G = .002$, indicating stronger SOA effects (presumably due to response grouping) with larger auditory scenes in task 2 (6 and 8, see Fig. 7). However, there was no main effect of set size on the response times of task 1, F(2.70,188.96)=0.60, $\textit{MSE} = 8,013.40$, p=.596, $\hat{\eta }^2_G = .000$. Target presence (in task 2) did not affect response times of task 1, F(1,70)=0.78, $\textit{MSE} = 7,078.59$, p=.381, and there was also no interaction between target presence and SOA, F(2.66,186.54)=1.59, $\textit{MSE} = 7,811.40$, p=.198. However, it appears that the auditory set-size effect on task 1 response times was stronger on the target-absent trials, as indicated by a set size × target presence interaction, F(2.78,194.75)=3.09, $\textit{MSE} = 7,919.26$, p=.031. Moreover, there was also a significant three-way interaction on task 1 response times, F(7.38,516.92)=3.17, $\textit{MSE} = 8,055.96$, p=.002, suggesting that the SOA effect at the larger set sizes was also restricted to trials on which the target was absent from the auditory scene in task 2.

Figure 8 illustrates the error rates and the response times in the *auditory search task 2* of the dual-task paradigm. For the *error rates* in task 2, there was a significant main effect of auditory set size, F(2.72,190.27)=3.63, $\textit{MSE} = 0.01$, p=.017, $\hat{\eta }^2_G = .003$, with slightly more incorrect responses with larger auditory scenes. However, alpha-corrected planned contrasts (Benjamini & Hochberg, 1995) revealed no reliable increase from set size 2 to 4, t(70)=2.03, $p_\mathrm {\scriptstyle BH(3)} = .073$, 4 to 6, t(70)=-0.54, $p_\mathrm {\scriptstyle BH(3)} = .590$, and 6 to 8, t(70)=2.01, $p_\mathrm {\scriptstyle BH(3)} = .073$. Task 2 errors were not subject to a main effect of SOA, F(2.95,206.38)=2.07, $\textit{MSE} = 0.01$, p=.107, $\hat{\eta }^2_G = .002$, and there was also no interaction between set size and SOA interaction, F(7.77,543.71)=0.35, $\textit{MSE} = 0.01$, p=.943, $\hat{\eta }^2_G = .001$. However, there was a significant main effect of target presence, F(1,70)=6.90, $\textit{MSE} = 0.01$, p=.011, $\hat{\eta }^2_G = .003$, with slightly more errors on target-present trials (see Fig. 8). Target presence did not interact with set size, F(2.77,193.87)=0.28, $\textit{MSE} = 0.01$, p=.827, $\hat{\eta }^2_G = .000$ or SOA, F(2.90,203.30)=1.19, $\textit{MSE} = 0.01$, p=.315, $\hat{\eta }^2_G = .001$, and there was no three-way interaction, F(7.58,530.60)=0.60, $\textit{MSE} = 0.01$, p=.773, $\hat{\eta }^2_G = .001$.

For the *response times* in the auditory search task 2, there was again a significant main effect of set size, F(2.80,195.74)=10.26, $\textit{MSE} = 15,216.96$, p<.001, $\hat{\eta }^2_G = .002$, with longer search times in larger auditory scenes indicating attentional capacity limitations during auditory scene analysis. Planned contrasts (alpha-corrected, Benjamini & Hochberg, 1995) revealed that — though not reaching statistical significance — response times tend to increase from set size 2 to 4, t(70)=2.21, $p_\mathrm {\scriptstyle BH(3)} = .080$, and from set size 6 to 8, t(70)=1.97, $p_\mathrm {\scriptstyle BH(3)} = .080$, whereas there is less evidence for a difference between the intermediate set sizes 4 and 6, t(70)=1.42, $p_\mathrm {\scriptstyle BH(3)} = .161$. There was also a significant SOA effect on response times, F(1.33,92.89)=482.02, $\textit{MSE} = 96,700.90$, p<.001, $\hat{\eta }^2_G = .220$, indicating capacity limitations of central attention at the response selection stage. Planned contrasts revealed that response times between all SOA conditions differed from each other (p<.001). Importantly, however, there was no significant interaction between SOA and set size for the response times, F(7.05,493.20)=1.50, $\textit{MSE} = 13,926.75$, p=.165, $\hat{\eta }^2_G = .001$, indicating that auditory search could again not be absorbed into the slack at the short SOAs (see Fig. 8). There was also a significant effect of target presence on the response times in the auditory search task, F(1,70)=126.58, $\textit{MSE} = 47,795.35$, p<.001, $\hat{\eta }^2_G = .027$, with longer response times when the target was absent (similar to the single-task results, see Fig. 8). Interestingly, there was also a significant interaction between target presence and set size, F(2.97,207.60)=3.98, $\textit{MSE} = 10,526.23$, p=.009, $\hat{\eta }^2_G = .001$, suggesting that the increase in auditory search times with the size of the auditory scene was more evident on trials in which the search target was absent. However, separate two-way ANOVAs revealed significant auditory set-size effects on both target-present, F(2.67,187.11)=2.97, $\textit{MSE} = 13,771.02$, p=.039, $\hat{\eta }^2_G = .001$ and target-absent trials, F(2.94,205.76)=12.21, $\textit{MSE} = 12,573.08$, p<.001, $\hat{\eta }^2_G = .004$. Finally, while there was no interaction between SOA and target presence, F(2.79,195.31)=0.35, $\textit{MSE} = 12,348.37$, p=.773, $\hat{\eta }^2_G = .000$, there was a significant three-way interaction between set size, SOA and target presence, F(6.74,471.82)=2.99, $\textit{MSE} = 14,440.75$, p=.005, $\hat{\eta }^2_G = .001$, indicating that there might be more evidence of a set size × SOA interaction on target-present trials than on target-absent trials (see Fig. 8). However, separate two-way ANOVAs revealed no significant interactions for both target-present, F(6.39,447.18)=1.12, $\textit{MSE} = 15,419.33$, p=.348, $\hat{\eta }^2_G = .001$, and target-absent auditory search times, F(7.29,510.62)=3.38, $\textit{MSE} = 13,291.59$, p=.001, $\hat{\eta }^2_G = .003$. Hence, even on target-present trials, there is no statistical evidence that auditory search can be performed in parallel with the central response selection stage.

**Fig. 9:**
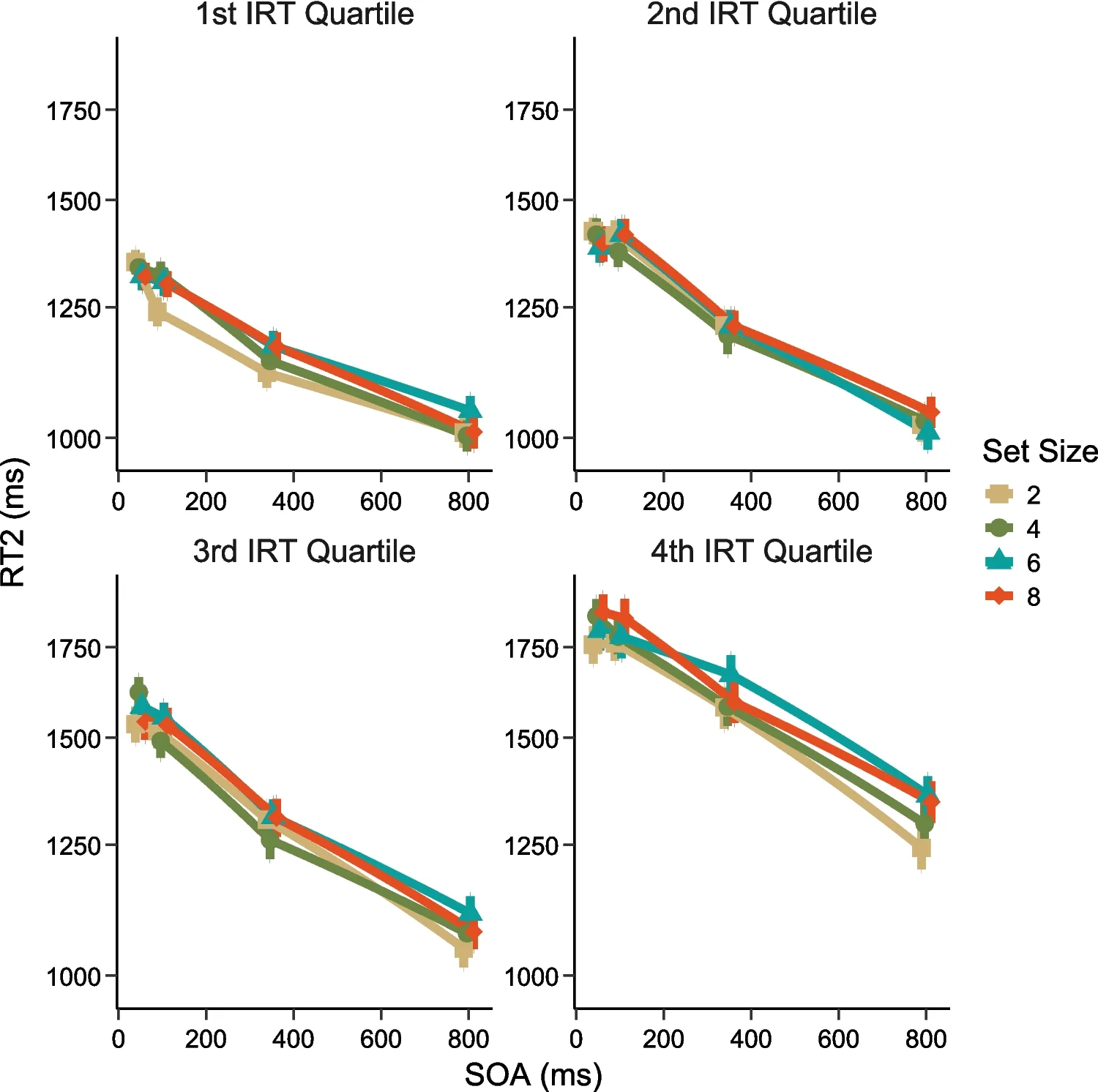
IRT Analysis showing similar effects of auditory set size on auditory search times (task 2) with short and long IRTs in Experiment 2

It was also tested whether the effects of auditory set size in Experiment 2 were influenced by response grouping. As in Experiment 1, the additional analysis of IRT quartiles revealed a significant main effect of the IRT quartile, F(1.40,97.97)=59.93, $\textit{MSE} = 207,553.68$, p<.001, $\hat{\eta }^2_G = .061$, with reduced response times in the short IRTs (see Fig. 9). In contrast to Experiment 1, there was a significant interaction between SOA and IRT quartile, F(6.43,449.85)=69.88, $\textit{MSE} = 24,865.27$, p<.001, $\hat{\eta }^2_G = .040$, but no interaction between set size and IRT quartile, F(7.51,525.60)=0.79, $\textit{MSE} = 16,866.58$, p=.600, $\hat{\eta }^2_G = .000$. This indicates that auditory search times may have been less affected by response grouping compared to Experiment 1, potentially due to the longer durations of the auditory scenes. On the other hand, the SOA effect appears slightly reduced at short IRTs, suggesting that response grouping may have affected the central response selection stage more than auditory search. Importantly, the analysis revealed no three-way interaction between SOA, set size and IRT quartile, F(16.65,1165.45)=1.41, $\textit{MSE} = 21,723.94$, p=.124, $\hat{\eta }^2_G = .002$, supporting the conclusion that the auditory set-size effects were equivalent across different SOAs, regardless of the IRT. Hence, sequential processing of auditory and central attention was again not contingent on response grouping.

### Discussion

Experiment 2 confirmed the capacity limitations of auditory attention that were observed in Experiment 1, but with slightly more complex and longer auditory scenes containing between two and eight unique animal sounds of 2-s duration. In comparison to the shorter sound durations from Experiment 1 (cut to 200 ms), these longer and more realistic sounds led to slightly longer auditory search times, in particular under dual-task conditions (1100–1600 ms compared to 1000–1400 ms in Experiment 1). Importantly, however, both error rates and response times again increased with the number of sounds contained in the scene both under single- and dual-task conditions, suggesting attentional capacity limitations and thus serial processing of individual sounds during auditory scene analysis. In the dual-task paradigm, these set-size effects were again found to be independent of the SOA between the two tasks, thus indicating that auditory search time cannot be absorbed into the slack at the very short SOAs. In fact, the processing demands of the visual task 1 were also increased compared to Experiment 1 (using a more complex visual color + shape discrimination task) in order to prolong the slack time. Nevertheless, in contrast to previously reported evidence on visual attention (e.g., Reimer & Schubert, 2019), this increase in task 1 difficulty did not eliminate the auditory set-size effects at short SOAs, suggesting that auditory search and response selection rely on common capacity limitations of central attention.

## General discussion

The goal of the present study was to (1) revisit the capacity limitations of auditory attention during auditory scene analysis (i.e., auditory search) and (2) test the assumed independence of auditory search processes from central attention – as indicated by a reduced set-size effect at short SOAs.

Across two online experiments, it was found that both response times and error rates in the auditory search task increased with the complexity of the auditory scene (i.e., the number of sounds). The auditory set-size effects in the single task indicate that attention is allocated sequentially to the different sound sources in an auditory scene similar to visual conjunction or spatial configuration search requiring the sequential allocation of attention to different visual objects (e.g., Treisman & Gelade, 1980; Wolfe et al., 2010). However, in contrast to typical visual search tasks, response times in auditory search did not increase linearly with set size. The strongest increase in response times was found from set size 1 to set size 2 (193 ms; Experiment 1), whereas the increase from set size 2 to 4 was much smaller (32 ms in Exp. 1 and 21 ms in Exp. 2). Further increases in set size led to even smaller increments in response times (6 ms / 12 ms from set size 4 to 6 in Exp. 1/2, respectively, and 3 ms from set size 6 to 8 in Exp. 2). This suggests that auditory search may use more efficient search processes leading to reduced costs at larger set sizes (e.g., a non-exhaustive hierarchical search mechanism allowing for some degree of parallel processing of different sounds; similar to response time increasing logarithmically with the number of items held in memory during visual hybrid search, Cunningham & Wolfe, 2014; Wolfe, 2012).

In addition to the auditory set-size effects, the dual-task also revealed longer auditory search times at short SOAs between the two tasks. This demonstrates the capacity limitations of central attention, presumably due to a bottleneck at the response-selection stage. However, in both experiments, the magnitude of auditory set-size effects did not depend on the SOA, suggesting that auditory search time cannot be absorbed into the slack. In other words, auditory scene analysis appears to be subject to the same central processing bottleneck that causes the PRP effect at the response selection stage. In contrast to some evidence from visual attention (e.g., Reimer & Schubert, 2019), the present study therefore indicates that auditory attention cannot operate in parallel with central attention due to shared capacity limitations. Here, the results also deviate from a previous study on auditory attention in the dual-task paradigm, which did reveal some reduction of auditory set-size effects at short SOAs when the processing demands of task 1 were high (Kattner & Reimer, 2020). However, a similarly or even more demanding visual discrimination task was used in the present study, suggesting that slack time probably was sufficiently long to absorb auditory search time. Given that the auditory set-size effects were extremely small in the previous study, it is more likely that the absence of set-size effects at some SOA conditions was due to the small effect sizes and lower statistical power of that experiment (*N* = 31 in Exp. 2, Kattner & Reimer, 2020) compared to the present experiments.

Admittedly, the set-size effects in the present Experiment 2 were smaller overall compared to Experiment 1, mainly due to the absence of the “auditory scenes” of set size 1, so it could be argued whether the set-size effects in Experiment 2 may just have been too small to observe a modulation by SOA. However, the dual-task paradigm of Experiment 2 revealed larger set-size effects on trials in which the target was absent, potentially due to the longer search times overall in the case of auditory scenes that did not contain the target sound. Importantly, even these larger set-size effects did not depend on the SOA, suggesting that exhaustive search of an auditory scene without the target depends on central attention. For the more efficient (faster) auditory search processes in case of a present target, the auditory set-size effects tend to become smaller at short SOAs (Fig. 8), but there was still no statistical evidence for search times to be absorbed into the slack of central processing. In both experiments, it was investigated whether auditory set-size effects were influenced by strategic response grouping. In particular, in Experiment 1, task 1 response times were found to increase with auditory set size in task 2, indicating that task 1 response selection may have been postponed until after completion of the auditory search task. To test whether auditory search was affected by response grouping, response times in task 2 were compared between trials with short and long delays between the two responses (IRTs) – with short IRTs typically indicating more response grouping. Importantly, it was found that auditory search times increased with set size even with very short IRTs (first quartile – though the set-size effect appears to be smaller compared to the higher quartiles), and the set-size effect did not depend on the SOA across all IRT quartiles. This suggests that auditory scene analysis (a) depended on the central processing bottleneck (i.e., auditory search time could not be absorbed into the slack) and (b) was not affected by strategic response grouping in Experiment 1. There was less indication of strategic response grouping in Experiment 2, since task 1 response times increased with the set size in task 2 only on trials without the auditory target. The IRT analysis also confirmed that the independent effects of set size and SOA on auditory search times did not differ between the quartiles of the IRT distribution, indicating again that auditory search was subject to the central processing bottleneck, regardless of strategic response grouping.

Taken together, the results tell us that the attentional guidance during auditory scene analysis is subject to the capacity limitations of central attention and cannot operate in parallel with other attention-demanding processes such as response selection. Specifically, across two experiments, both error rates and response times increased with the complexity of an auditory scene in which a target was to be detected, under both single-task and dual-task conditions. In the dual-task paradigm, these auditory set-size effects were not reduced at short SOAs between the two tasks, suggesting that auditory search time could not be absorbed by the slack that emerges due to the central processing bottleneck (i.e., the PRP effect). In other words, the goal-directed guidance of attention through auditory scene analysis cannot begin before the previous task’s response selection is finished, thus producing additive effects of increased auditory set size and reduced SOA on auditory search times. The study also demonstrates that it is possible to induce and measure effects of both auditory and central attention in online studies, provided that participants use appropriate headphones. The magnitude of both the PRP effect (increase in response times to auditory scenes at short SOAs) and the effect of auditory set size was very much comparable to (or even larger than) the effects reported in previous studies using more controlled laboratory settings (Kattner & Reimer, 2020; Reimer & Schubert, 2019).

## Data Availability

See Open Practices and Data Availability Statement.

## Footnotes

1 Based on Experiment 1: $F(2.76, 124.31)=7.04; M_3=923; SD_3=119; M_7=941; SD_7=131$

## Code availability

See Open Practices and Data Availability Statement.

## Declarations

### Open Practices Statement

Stimulus materials, aggregated data, and the full code to analyze the data and reproduce this manuscript are openly available in this repository: https://osf.io/k7msx/overview?view_only=39912094467640b9b60c67a3869ec2ce. This study was not preregistered.
